# SLC4A11 and the Pathophysiology of Congenital Hereditary Endothelial Dystrophy

**DOI:** 10.1155/2015/475392

**Published:** 2015-09-16

**Authors:** Sangita P. Patel, Mark D. Parker

**Affiliations:** ^1^Department of Ophthalmology, Ross Eye Institute, School of Medicine and Biomedical Sciences, The State University of New York at Buffalo, 1176 Main Street, Buffalo, NY 14209, USA; ^2^SUNY Eye Institute, Buffalo, NY 14214, USA; ^3^Research Service, Veterans Administration Western New York Healthcare System (VAWNYHS), Building 20, 3495 Bailey Avenue, Buffalo, NY 14215, USA; ^4^Department of Physiology and Biophysics, The State University of New York at Buffalo, 124 Sherman Hall, Buffalo, NY 14214, USA

## Abstract

Congenital hereditary endothelial dystrophy (CHED) is a rare autosomal recessive disorder of the corneal endothelium characterized by nonprogressive bilateral corneal edema and opacification present at birth. Here we review the current knowledge on the role of the *SLC4A11* gene, protein, and its mutations in the pathophysiology and clinical presentation of CHED. Individuals with CHED have mutations in *SLC4A11* which encodes a transmembrane protein in the SLC4 family of bicarbonate transporters. The expression of SLC4A11 in the corneal endothelium and inner ear patterns the deficits seen in CHED with corneal edema and hearing loss (Harboyan syndrome). *slc4a11*-null-mouse models recapitulate the CHED disease phenotype, thus establishing a functional role for SLC4A11 in CHED. However, the transport function of SLC4A11 remains unsettled. Some of the roles that have been attributed to SLC4A11 include H^+^ and NH_4_^+^ permeation, electrogenic Na^+^-H^+^ exchange, and water transport. Future studies of the consequences of SLC4A11 dysfunction as well as further understanding of corneal endothelial ion transport will help clarify the involvement of SLC4A11 in the pathophysiology of CHED.

## 1. Introduction

Congenital hereditary endothelial dystrophy (CHED) is a rare disorder of the corneal endothelium with an early onset of corneal edema. Original classifications of CHED described two forms [1]. CHED1 was an autosomal dominant disease and presented with progressive corneal clouding beginning within the first few years of life. CHED2 was an autosomal recessive disease and presented with corneal clouding at birth or in the immediate newborn period. Recent genetic analyses and review of clinical findings now confirm that the previously termed autosomal dominant CHED (originally CHED1) is a form of posterior polymorphous corneal dystrophy with early and severe corneal edema [2]. It is thus no longer considered in the category of CHED (i.e., the entity CHED1 is now classified with posterior polymorphous corneal dystrophy). Based on the 2015 update to the International Classification of Corneal Dystrophies, the term “CHED” now exclusively refers to autosomal recessive CHED (originally CHED2) [3]. This review adopts this new classification and discusses only the autosomal recessive disease, henceforth called “CHED.” Mutations in SLC4A11, a transmembrane protein in the family of bicarbonate transporters, are present in the majority of CHED cases studied. This review focuses on the role of SLC4A11 in the pathophysiologic mechanisms and clinical presentation of CHED.

## 2. Mutations in* SLC4A11*

**Figure 1:**
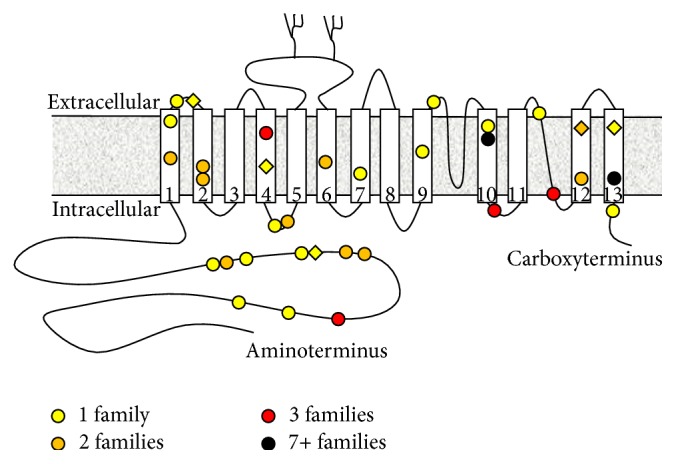
Cartoon of SLC4A11 protein topology showing the location of CHED-linked missense mutations. Approximately half of the individuals identified with CHED-linked mutations in SLC4A11 carry homozygous nonsense, frameshift, deletion, or splice-site mutations (not shown) that are predicted to result in the loss of active SLC4A11 protein. The other half carry homozygous missense mutations (colored circles) that are predicted to alter the protein sequence of SLC4A11 protein, noting the site of residues that are presumably important for the correct folding and activity of SLC4A11. Six individuals with CHED carry compound heterozygous (CH) mutations in SLC4A11: the location of missense mutations that have only been described in these individuals, and not in homozygous form, is marked with colored diamonds. The color of the circles and diamonds denotes the number of cases in which the mutation has been observed. Moving from amino- to carboxyterminus, the single cases (yellow) are R125H, E143K, S232N (CH with R329X), R233C, T262I, T271M, G394R, E399K, T401K (CH with L473R), L473R (CH with T401K), R488K, C611R, G709E, H724D, T754M, R804M, M856V (CH with S213P), and L873P. Sites mutated in two instances (orange) are R209W, S213L (plus CH S213P/M856V), A269V, C386R, G417R, G4181D, S489L, T584K (plus CH T548K/R112X), T833M, and L843P (both instances in CH form with frameshift mutations). Sites mutated in three instances (red) are A160T, G464D, P773L (including CH P773L/R112X), and V824M. Note that homozygous inheritance of A160T has also been observed in one unaffected individual and thus may not be the exclusive cause of CHED in these individuals [4]. Sites mutated in seven or more instances (black) are R755Q/R (including five instances of R755Q, one CH case of R755Q/R875X, and four instances of R755W) and R869C (seven instances). References: [4–16].

The first clue to the origin of CHED came from the use of mapping techniques that enabled researchers to link autosomal recessive inheritance of CHED to genetic markers within the 20p13 chromosomal locus [5, 22–25]. This assignment eventually led to the identification of mutations in the* SLC4A11* gene as the genetic basis of CHED [5].* SLC4A11* encodes a membrane protein that was originally termed “BTR1” (Bicarbonate Transporter Related protein 1): a name that reflects its membership in the SLC4 family of bicarbonate transporting membrane proteins [26]. Despite the name, the disparate amino acid sequence of SLC4A11 segregates it from the family. Perhaps, as a consequence, robust HCO_3_^−^ transport is not among the numerous molecular actions that have been attributed to SLC4A11 [17, 27]. At the time of writing, mutations in* SLC4A11* have been described in more than one hundred individuals with CHED (Figure 1) and signs of CHED have been recapitulated in several strains of* slc4a11*-null mice [18, 19, 28]. However, there is still much to be learned about the molecular action and the role of SLC4A11 in the healthy and diseased cornea.

## 3. Clinical Phenotype and Molecular Expression

The* SLC4A11* gene, which encodes at least three products (SLC4A11-A, -B, and -C), is expressed in a wide variety of cell types including corneal endothelium, spiral ligament fibrocytes of the inner ear, and various renal epithelia [5, 18, 19, 21, 29]. The molecular expression pattern of SLC4A11 correlates with the observed clinical phenotype for autosomal recessive CHED.

### 3.1. Corneal Endothelium

**Figure 2:**
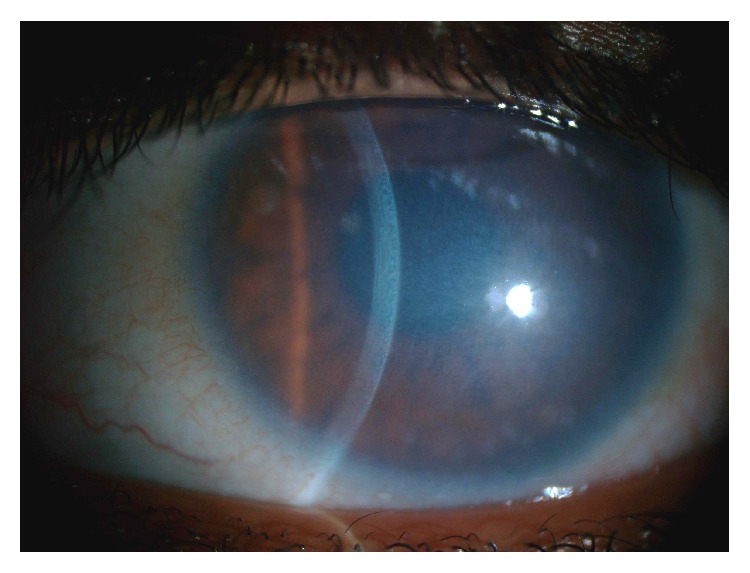
Clinical photograph of CHED demonstrating bluish-gray ground glass appearance. The slit beam highlights the uniform thickening of the cornea. Note the lack of corneal vascularization and inflammation. (Photograph courtesy of Arif O. Khan, Division of Pediatric Ophthalmology, King Khaled Eye Specialist Hospital, Riyadh, Saudi Arabia.)

Disease of the corneal endothelium is the hallmark of CHED with corneal edema and opacification presenting at birth or shortly thereafter. The degree of opacification varies from mild to severe with a bluish-gray ground glass appearance (Figure 2). The opacification does not typically progress or regress. The cornea is uniformly thickened 2-3 times normal. Nystagmus of varying degrees and amblyopia may be present in individuals with more severe opacification. Photophobia, epiphora, and inflammation are not prominent features. (In contrast, the previously termed CHED1, now posterior polymorphous corneal dystrophy, presents with progressive corneal edema and opacification, typically not present at birth. Photophobia and epiphora are more common.) Primary disease of the corneal endothelium is the culprit for edema in CHED. The normal hexagonal endothelial mosaic is altered or absent. When endothelial cells can be visualized by specular or confocal microscopy, the cells are attenuated and fibrotic. Thickening of the endothelial basement membrane, Descemet's membrane, is visible by slit lamp examination. Two of three mouse models with disruptions in* slc4a11* have also replicated the corneal defects seen in humans [29–31]. These mice have progressive corneal swelling, doubling the thickness of the cornea, and thickening of Descemet's membrane [30, 31]. In these mouse models, the endothelium is present but the endothelial cells of older mice are swollen, distorting the hexagonal array, and exhibit vacuolization indicating cell distress [30, 31].

### 3.2. Inner Ear

In the mouse inner-ear, slc4a11 is expressed in the vestibular labyrinth and in fibrocytes underlying the stria vascularis [29, 30]. The stria vascularis is responsible for formation of endolymph and vestibular labyrinth for transduction of signals for hearing and balance. As may be expected from this expression pattern, high-frequency hearing loss is also a feature of CHED. Harboyan syndrome describes the condition of sensorineural hearing loss in the setting of CHED [32]. Although it has been described as an entity separate from CHED, a recent study suggests that some degree of hearing loss may develop in all individuals with time [33, 34]. Given the progressive nature of hearing loss in Harboyan syndrome, it may be missed in some individuals if tested at too young age. To date, the hearing loss has never been documented in the prelingual period. Progressive hearing loss is also recapitulated in mice with disrupted* slc4a11*, even in the strain of mouse that did not exhibit any CHED-like ocular signs [29, 30].

### 3.3. Kidney

Slc4a11 is expressed in mouse kidney and has been detected in the proximal tubules, the thin descending limb of the loop of Henle, and the collecting ducts [30, 35]. Two mouse models of CHED with disruptions of* slc4a11* have a urine concentrating defect resulting in decreased urine osmolarity and corresponding decrease in all urine electrolyte concentrations [30, 31]. Interestingly, there is only one report evaluating kidney function in one human with CHED and no defect was found. The older age of this individual (55 years old) suggests that any potential progressive defect would have been detected if present compared to evaluation in a younger individual [36].

### 3.4. Trabecular Meshwork

There are several case reports of glaucoma presenting in patients with CHED. However, to date, there are no reports on the expression of SLC4A11 in the trabecular meshwork and aqueous outflow pathways of the eye. Review of published cases suggests a tenuous association. One series reported three cases of glaucoma with CHED [37]. The first case included a family history of congenital glaucoma and the subject also had partial aniridia with ectropion uveae. In the second case, iris vascularization was noted as well as heavy vascularization of the anterior stroma upon histological examination of the excised cornea. In the third case, the subject had partial aniridia. A subject in another case report of CHED with glaucoma also had iris hypoplasia [38]. Review of these published cases suggests the involvement of more global anterior segment dysgenesis in addition to CHED. Faced with the potential diagnosis of glaucoma in CHED, careful examination should be performed to consider the diagnosis of posterior polymorphous corneal dystrophy (formerly autosomal dominant CHED1) for which glaucoma is a more common codiagnosis (15%) [3]. While the common origin of the corneal endothelium and anterior chamber angle from neural crest would support the association of glaucoma and CHED, SLC4A11 expression has not been demonstrated in aqueous outflow structures of the eye.

## 4. The Molecular Actions of SLC4A11

**Figure 3:**
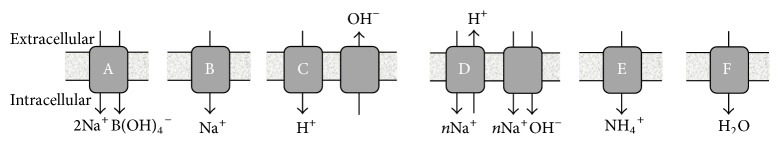
Suggested molecular actions of the SLC4A11 protein. A: Electrogenic sodium/borate cotransporter [17]. B: conductive sodium permeation pathway [17]. C: conductive proton influx permeation pathway, which is thermodynamically equivalent to a hydroxyl efflux pathway [17, 18]. D: coupled electrogenic *n*Na/H exchange, which is equivalent to electrogenic *n*Na-OH cotransport (*n* > 1) [19, 20]. E: NH_4_^+^ permeation pathway [20]. F: H_2_O permeation pathway [21].

SLC4A11 dysfunction clearly plays a key role in CHED pathogenesis, but little is known of its normal function. Several molecular actions have been assigned to SLC4A11, yet which of these are of physiological or pathophysiological importance remains to be determined (Figure 3). The first study to address the action of SLC4A11 reported that human SLC4A11 expressed in the HEK293 human-kidney-derived cell line formed Na^+^ and H^+^ (or OH^−^) channel [27]. Na^+^ and/or H^+^/OH^−^ transport mediated by SLC4A11 has subsequently been observed in other mammalian cell lines [20, 31] and in cultured bovine corneal endothelial cells [17]. SLC4A11 has also been noted to enhance NH_4_^+^ permeability in HEK293 cells [31]. Intriguingly, SLC4A11 can also act like an aquaporin, enhancing cellular water permeability [21]. An initial report that SLC4A11 was an electrogenic Na^+^-coupled borate cotransporter (therefore renamed “NaBC1”) [27] is controversial as others have been unable to detect evidence for any borate-dependent action of SLC4A11 [17, 20, 21, 31]. With all of these observations on SLC4A11 action but no consensus, the possibility remains that SLC4A11 has another function, yet to be determined.

## 5. The Role of SLC4A11 in the Cornea

**Figure 4:**
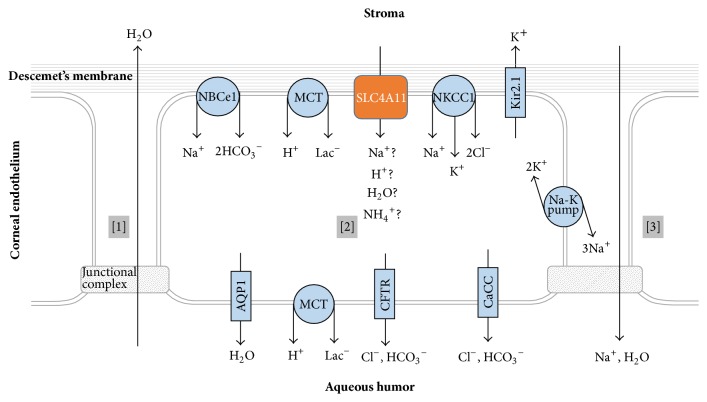
Transporters and channels that support corneal pump function. [1] Water is drawn from the aqueous humor into the stroma. [2] Endothelial cells secrete ions into the aqueous humor creating osmotic gradient that [3] draws fluid back into the aqueous humor.

Immunohistochemical studies reveal the presence of SLC4A11 in the corneal endothelium of humans, rats, and mice [18, 21, 28, 29]. In the corneal endothelium of mice, slc4a11 is present exclusively in the basolateral membrane [18, 21]. The purpose of the endothelial layer is to prevent stromal edema by countering the osmotically driven movement of water from the aqueous humor into the collagen matrix of the stroma. The endothelial cell layer is leaky due to its high paracellular permeability. Thus, rather than prevent the movement of water into the stroma, endothelial cells draw water out of the stroma coupled to secretion of ions from the stroma to the aqueous humor. This “pump-leak” mechanism, recently reviewed in [39], is represented in Figure 4. The usefulness of SLC4A11 to normal corneal function has yet to be fully elucidated, in part because its* in vivo* action is uncertain. If SLC4A11 functions like an aquaporin to mediate H_2_O flux, then it could promote transcellular (stroma to aqueous humor) water flux in concert with aquaporin 1 [21]. If SLC4A11 functions in Na^+^-H^+^/OH^−^ transport, then it could support the “pump” mechanism by modulating intracellular pH, volume, or membrane potential [17, 20].

## 6. The Contribution of SLC4A11 Dysfunction to CHED

Humans harboring* SLC4A11* mutations (or mice with a disrupted* slc4a11* gene) exhibit contrasting corneal endothelial phenotypes which may help advance our understanding of SLC4A11 dysfunction in CHED. In humans, the corneal endothelial monolayer is present and dystrophic or is absent with only rare fibrotic-appearing, atrophic endothelial cells present [6, 40–42]. In contrast,* slc4a11*-null mice have a corneal endothelial monolayer of cells present [30, 31]. However, disease is evidenced in these cells by the presence of vacuolization, gradual decrease in endothelial cell density, and loss of hexagonal cell morphology. Despite the presence of a corneal endothelial monolayer, these mice have corneal edema with corneal thickness increasing with age, thus arguing that the deficit in CHED lies with the efficacy of endothelial cell function.

The embryology of corneal endothelium and Descemet's membrane formation also supports the argument of disruption of endothelial cell function. The dysfunction occurs postnatally. The first wave of migration of neural crest cells from the rim of the invaginating optic cup forms the corneal endothelial monolayer and trabecular meshwork [43]. Two factors indicate that the defect in CHED is not with this initial migration. First, if the defect in CHED were in this initial migration, one might expect a higher incidence of glaucoma (due to comigration of cells forming trabecular meshwork) than is currently observed in individuals with CHED. Secondly, corneal endothelial cells secrete the anterior banded zone of Descemet's membrane beginning around the 3rd month and continuing through the 8th month of gestation [44]. The anterior banded zone is absent in conditions such as Peter's anomaly with defects in neural crest cell migration to form anterior segment structures [45, 46]. In contrast, the anterior banded zone is present in CHED with either normal or thickened morphology, thus indicating that the endothelial cells were present and functional during that period of development [40, 41]. During early postnatal development, the corneal endothelium begins formation of the posterior nonbanded zone (PNBZ) of Descemet's membrane. The PNBZ continues to thicken throughout life [47]. In diseases with dysfunctional endothelium, the PNBZ can merge with an abnormal posterior collagenous layer that is secreted by the endothelial cells. In humans with CHED, the PNBZ has variable thickness (thin or thick) with or without the presence of a posterior collagenous layer [40–42]. This variability in thickness of the posterior portion of Descemet's membrane may reflect the variability in timing of demise of the corneal endothelial cells.

There are numerous ways a defective membrane transport protein could contribute to a disease state, the most obvious being loss of transport function. Others include loss of protein* per se* (and thence loss of docking sites for dependent interacting-proteins), cell stress due to the accumulation of misfolded transport protein, and maladaptive compensatory changes in the expression of other gene products. Many of the CHED mutations recapitulated in heterologous systems are predicted to generate a misfolded SLC4A11 protein and have been shown to accumulate in intracellular compartments when expressed in cultured cells [5]. As mentioned above, endothelial cells do exhibit signs of stress with vacuolization and deposition of the posterior layer of Descemet's membrane. However it does not seem that the anticipated stress from accumulation of misfolded protein is the sole driving force behind the manifestation of CHED. One CHED-linked mutation exhibits loss of H_2_O and H^+^/OH^−^ transport in model systems without any deleterious effect upon SLC4A11 protein expression [20, 21]. Moreover, corneal edema is recapitulated in a strain of* slc4a11*-null mouse that is predicted to express no misfolded slc4a11 protein product [31]. Thus, whatever its molecular action may be, the pathology behind CHED seems to involve a loss of SLC4A11-mediated support of “pump” function.

## 7. Conclusion

Current advances in genetic testing for individuals with CHED have narrowed the spectrum of disease to mutations in a single gene,* SLC4A11*. Mouse models of CHED with deficits in slc4a11 expression recapitulate many of the features of the disease and will allow us to better understand its development and pathophysiology. The functional role of SLC4A11 in the corneal endothelium is unsettled yet it likely impacts corneal endothelial ion transport as part of the “pump” mechanism to maintain corneal clarity.
